# Detection of Blood Stains

**Published:** 1869-10

**Authors:** 


Detection of Blood Stains.—The detection of blood in old, and often minute stains on clothing, wood, metal, &c., can now be made with absolute certainty. The crystals of hæmatine which can be separated from the slightest traces of blood can always be recognized by means of the microscope—but to decide to what species, whether man or lower animal the blood belongs, is a question attended with great difficulty and uncertainty. In view of this fact, Neumann has recently subjected the question to a rigid and exhaustive examination and has published his results in a book. The work contains twenty-three superbly executed colored plates in which nineteen different kinds of blood are represented, in which the differences of the microscopic examination are displayed in a wonderfully clear manner.

Neumann recommends to moisten the blood stain with distilled water and to heat to about 60 degrees Fahrenheit on the glass side of the microscope. In this way microscopic pictures of human and animal blood are obtained of such dissimilarity that human blood can readily be distinguished from that of any other animal. The author explains in what way this is done and gives ample illustration.

